# Overexpression of microRNA-223 regulates the ubiquitin ligase FBXW7 in oesophageal squamous cell carcinoma

**DOI:** 10.1038/bjc.2011.509

**Published:** 2011-11-22

**Authors:** J Kurashige, M Watanabe, M Iwatsuki, K Kinoshita, S Saito, Y Hiyoshi, H Kamohara, Y Baba, K Mimori, H Baba

**Affiliations:** 1 Department of Gastroenterological Surgery, Graduate School of Medical Sciences, Kumamoto University, 1-1-1 Honjo, Kumamoto 860-8556, Japan; 2 Department of Surgery, Medical Institute of Bioregulation, Kyushu University, 4546 Tsurumihara, Beppu 874-0838, Japan

**Keywords:** microRNA, FBXW7, ubiquitin ligase

## Abstract

**Background:**

F-box and WD repeat domain-containing 7 (FBXW7) is a cell cycle regulatory gene whose protein product ubiquitinates positive cell cycle regulators such as c-Myc, cyclin E, and c-Jun, thereby acting as a tumour-suppressor gene. This study focused on microRNA-223 (miR-223), which is a candidate regulator of *FBXW7* mRNA. The aim of this study was to clarify the clinical significance of miR-223 and FBXW7 in oesophageal squamous cell carcinoma (ESCC) patients, and to elucidate the mechanism by which FBXW7 is regulated by miR-223.

**Methods:**

The expression levels of miR-223 and the expression of FBXW7 protein was examined using 109 resected specimens to determine the clinicopathological significance. We also investigated the role of miR-223 in the regulation of *FBXW7* expression in ESCC cell lines in an *in vitro* analysis.

**Results:**

We found that miR-223 expression was significantly higher in cancerous tissues than in the corresponding normal tissues. There was a significant inverse relationship between the expression levels of miR-223 and FBXW7 protein. Moreover, patients with high miR-223 expression demonstrated a significantly poorer prognosis than those with low expression. On the basis of a series of gain-of-function and loss-of-function studies *in vitro*, we identified *FBXW7* as a functional downstream target of miR-223.

**Conclusion:**

Our present study indicates that high expression of miR-223 had a significant adverse impact on the survival of ESCC patients through repression of the function of FBXW7.

F-box and WD repeat domain-containing 7 (FBXW7) is the substrate recognition component of an evolutionarily conserved SCF (complex of SKP1, CUL1, and F-box protein)-type ubiquitin ligase complexes, which has been well characterised and shown to have important roles in regulating the stability of multiple oncoprotein substrates, including cyclin E, c-Myc, Notch, c-Jun, mammalian target of rapamycin, and MCL1 (Nakayama and Nakayama, 2006; Mao *et al*, 2008; Welcker and Clurman, 2008; Inuzuka *et al*, 2011; Wertz *et al*, 2011). Therefore, the altered expression of FBXW7 is recognised to be one of the major causes of carcinogenesis or cancer development. We have previously revealed that loss of *FBXW7* correlated with a poor prognosis in colon cancer (Iwatsuki *et al*, 2010). Similarly, low expression of *FBXW7* has been reported to be significantly associated with poor prognoses in glioma (Bredel *et al*, 2005; Hagedorn *et al*, 2007), gastric cancer (Yokobori *et al*, 2009), and breast cancer (Ibusuki *et al*, 2011). However, significance of downregulation of this molecule in oesophageal squamous cell carcinoma (ESCC) remains unknown.

Recent studies have revealed that microRNAs (miRNA) can act as oncogenes or tumour suppressors during the development and progression of cancers through sequence-specific binding to their mRNA targets (Ambros, 2004; Zamore and Haley, 2005; Meister, 2007). These miRNAs have an important role in a wide variety of complex biological processes, including cellular development and differentiation, but investigations have only begun to clarify their significance in carcinogenesis (Calin *et al*, 2004; Croce and Calin, 2005). Some researchers have noted that alterations in the miRNA expression profile strongly affect the progression of human tumours and the prognosis of the patients (Yanaihara *et al*, 2006; Bloomston *et al*, 2007; Schetter *et al*, 2008; Ueda *et al*, 2010). Although our previous study revealed that miR-21 is significantly overexpressed in ESCC (Hiyoshi *et al*, 2009), there have been few reports concerning the miRNA profiles in ESCC.

It was recently reported that microRNA-223 (miR-223) targets the *FBXW7* mRNA 3′-untranslated region, and that overexpression of miR-223 significantly reduces *FBXW7* mRNA levels, increases endogenous cyclin E protein and activity levels, and increases genomic instability (Xu *et al*, 2010). Nevertheless, to our knowledge, no study has been reported on the relationship between the expression levels of miR-223 and FBXW7 in clinical samples of solid tumours. However, no correlation between miR-223 and FBXW7 has yet been elucidated in ESCC.

In the present study, we examined the correlation between the expression levels of miR-223 and immunohistochemical staining for the FBXW7 protein in 109 consecutive ESCC samples, and we also investigated the prognostic significance of the expression of miR-223. Moreover, we identified *FBXW7* as a functional downstream target of miR-223 *in vitro*.

## Patients and methods

### Patients and tissue samples

Primary ESCC tissue samples and samples of matched normal oesophageal epithelium were obtained from 109 patients who underwent oesophageal resection without preoperative treatment in the Department of Gastroenterological Surgery, Kumamoto University Hospital from 2000 to 2007. Written informed consent was obtained from all patients. The clinicopathological characteristics, including age, gender, pathology, differentiation, and tumour-node metastasis classification were available for all patients. Survival was measured from the time of oesophageal resection and death was the endpoint. The patient prognoses were examined in March 2011. The median observation time for survival was 31 months and it ranged from 1 to 132 months. The study was approved by the medical ethics committee of Kumamoto University.

### ESCC cell lines

The human ESCC cell lines TE1, TE4, TE6, TE8, TE9, TE10, TE14, and TE15 were provided by the Cell Resource Center for Biomedical Research Institute of Development, Aging and Cancer, Tohoku University, Japan. All cells were grown in RPMI 1640 (Cambrex, East Rutherford, NJ, USA) supplemented with 10% fetal bovine serum (Sigma-Aldrich, St Louis, MO, USA), 2 mmol l^−1^ glutamine, 100 units of penicillin per ml, and 100 *μ*g of streptomycin per ml (Cambrex), and were incubated at 37°C in a humidified chamber supplemented with 5% CO_2_.

### miRNA isolation

The miRNAs were extracted from formalin-fixed, paraffin-embedded oesophageal tissues using a RecoverAll Total Nucleic Acid Isolation Kit for FFPE (Ambion, Austin, TX, USA), according to the manufacturer's instructions. The purity and concentration of all RNA samples were evaluated by their absorbance ratio at 260/280 nm determined with a NanoDrop ND-1000 spectrophotometer (NanoDrop Technologies, Rockland, DE, USA).

### Quantitative real-time reverse transcription-PCR (qRT–PCR)

The expression levels of miR-223 were determined by TaqMan qRT-PCR using TaqMan microRNA assay kits (Ambion, USA) according to the manufacturer's protocols, as described previously (Hiyoshi *et al*, 2009). The miR-223 expression was normalised to that of RNU6B, a small nuclear RNA. The expression levels of *FBXW7* were determined using primers and probes that were designed using the Universal Probe Library (Roche Diagnostics, Mannheim, Germany) following the manufacturer's recommendations. The primer sequences used for real-time PCR were as follows: *FBXW7* forward 5′-AAAGAGTTGTTAGCGGTTCTCG-3′, reverse 5′-CCACATGGATACCATCAAACTG-3′and universal probe #78; *18s rRNA* forward 5′-TGGAGGAGACGTTCCAGTGT-3′, reverse 5′-GATCTGTCCAGGCAGTCCTT-3′ and universal probe #17. All qRT–PCR reactions were run using a LightCycler 480 System II (Roche Diagnostics, USA). The relative amounts of miR-223 and *FBXW7* were measured using the 2^−ΔΔCT^ method. All qRT–PCR reactions were performed in triplicate.

### Immunohistochemical analysis

The immunohistochemical studies for FBXW7, c-Myc, and c-Jun were performed on formalin-fixed, paraffin-embedded surgical sections obtained from 109 patients with ESCC. Tissue sections of 5 *μ*m thickness were deparaffinized and pre-treated for antigen retrieval by autoclave heating in 10 mM sodium citrate buffer (pH 9.0) for 15 min. These sections were blocked for endogenous peroxidase activity with 3% H_2_O_2_ in methanol for 60 min and then washed in phosphate-buffered saline. The sections were incubated in primary mouse monoclonal anti-FBXW7 (1 : 100, Abnova Corporation, Taipei, Taiwan), mouse monoclonal anti-c-Myc (sc-40, 1 : 100, Santa Cruz Biotechnology, Fremont, CA, USA), or rabbit polyclonal anti-c-Jun (sc-1694, 1 : 50, Santa Cruz Biotechnology) antibody. Tissue sections were immunohistochemically stained using ENVISION reagents (ENVISION+ Dual Link System-HRP, Dako Cytomation, Glostrup, Denmark). All sections were counterstained with haematoxylin. The staining assessment was independently carried out by two authors (JK and YB) without any information about the patients’ clinicopathological characteristics or prognosis.

### Transfection of miRNA

The cells were transfected with 20 nM Pre-miR miRNA Precursor Molecule pre-223 (pre-miR-223) and 100 nM anti-miR miRNA inhibitor anti-223 (anti-miR-223) (Applied Biosystems) using the Lipofectamine 2000 transfection reagent (Invitrogen, Carlsbad, CA, USA), according to the manufacturer's instructions. The specificity of the transfection was verified using the Pre-miR miRNA Precursor Molecule Negative Control #1 (control pre-miR) and Anti-miR miRNA Inhibitors Negative Control #1 (control anti-miR) (Applied Biosystems). The expression levels of miR-223 and *FBXW7* were quantified 72 h after transfection, and the cells were used for a western blot analysis.

### Western blot analysis

To isolate the proteins, cells harvested from 6-well plates were washed once in phosphate-buffered saline and lysed in lysis buffer (Tris-HCl (pH 7.4) 25 mmol l^−1^, NaCl 100 mmol l^−1^, EDTA 2 mmol l^−1^, Triton X 1%, with 10 *μ*g ml^−1^ aprotinin, 10 *μ*g ml^−1^ leupeptin, and 1 mmol l^−1^ Na_3_VO_4_, 1 mmol l^−1^ phenylmethylsulfonylfluoride). Each protein sample (15 *μ*g) was resolved by sodium dodecyl sulphate–polyacrylamide gel electrophoresis, transferred onto a polyvinylidene difluoride membrane, and incubated with a monoclonal antibody against c-Myc (sc-40, 1 : 500, Santa Cruz Biotechnology), c-Jun (sc-1694, 1 : 500, Santa Cruz Biotechnology) or *β*-actin (1 : 2000; Sigma-Aldrich). The signals were detected by incubation with secondary antibodies labelled using the ECL Detection System (GE Healthcare, Little Chalfont, UK).

### Statistical analysis

All experiments were repeated at least three times. Continuous variables were expressed as the means±s.d. The relationship between the expression of miR-223, the FBXW7 protein, and the patient clinicopathological characteristics was analysed using Student's *t-*test or a *χ*^2^-analysis. The overall survival curves were plotted according to the Kaplan–Meier method, and the generalised log-rank test was applied to compare the survival curves. The findings were considered to be significant at a *P*-value <0.05. All statistical analyses were performed using the SPSS v. 13.0 software program (SPSS, Inc., Chicago, IL, USA).

## Results

### Clinicopathological significance of miR-223 in ESCC patients

**Figure 1:**
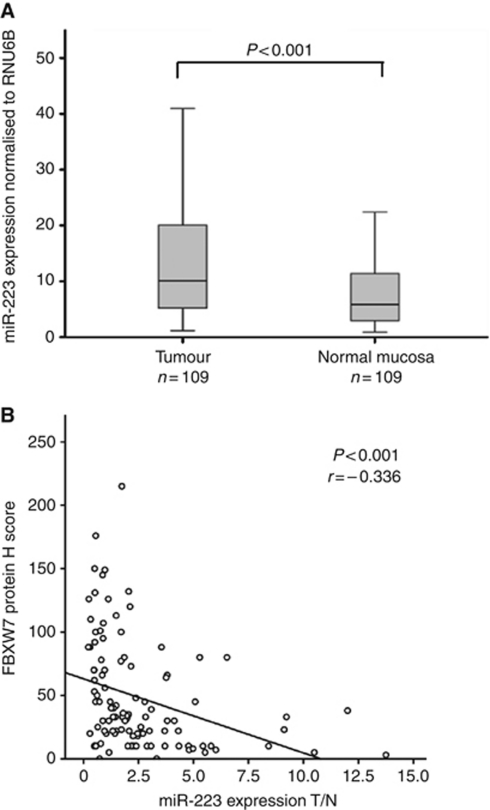
The correlation between the expression of miR-223 and the FBXW7 protein in ESCC patients. (**A**) The expression level of miR-223 in tumour tissue specimens was significantly higher than that in non-tumour tissues (*P*<0.001). (**B**) To evaluate the expression of FBXW7, the complete H score was semiquantitatively calculated (0–300). The relationship between miR-223 and FBXW7 in 109 clinical samples of ESCC indicated an inverse correlation (Pearson correlation, *r*=−0.336; *P*<0.01).

The expression levels of miR-223 were examined in 109 ESCC clinical samples using qRT–PCR, with quantified values used to calculate miR-223/U6B ratios. The mean expression levels of miR-223 in cancerous tissue specimens were significantly higher than those in non-cancerous tissues (*P*<0.001; Figure 1A). We divided the 109 ESCC patients into two groups according to the ratio of their cancer/normal tissue expression levels of miR-223, as ⩾1.0 or <1.0 for the cancer/noncancerous tissues expression levels of miR-223. There were 74 cases (67.9%) in the high miR-223 group and 35 cases (32.1%) in the low miR-223 expression group. The association between the patient clinicopathological characteristics and miR-223 expression is summarised in Table 1. There were significant differences in gender (*P*=0.008), tumour size (*P*=0.042), and depth of tumour invasion (*P*=0.030) between the groups.

### The relationship between the expression of miR-223 and immunohistochemical staining for the FBXW7 protein in ESCC tissues

A fragment of the *FBXW7* 3′-untranslated region contained three putative miR-223 binding sites as determined by a computational analysis using miRNA target prediction programs such as Target Scan (http://www.targetscan.org) and miRanda (http://www.microrna.org) (Supplementary Figure 1).

We examined the FBXW7 protein expression level by an immunohistochemical analysis in the samples from ESCC patients. To evaluate the FBXW7 expression, the complete H score was semiquantitatively calculated by summing the products of the percentage of cells stained at a given staining intensity (0–100) and the staining intensity score (0, none; 1, weak; 2, moderate; and 3, intense). We found an inverse correlation between the expression levels of miR-223 and FBXW7 in 109 clinical samples of ESCC. High levels of miR-223 were associated with low FBXW7 expression (Pearson correlation, *r*=−0.336; *P*<0.01; Figure 1B).

### The prognostic significance of miR-223 and FBXW7 in ESCC

**Figure 2:**
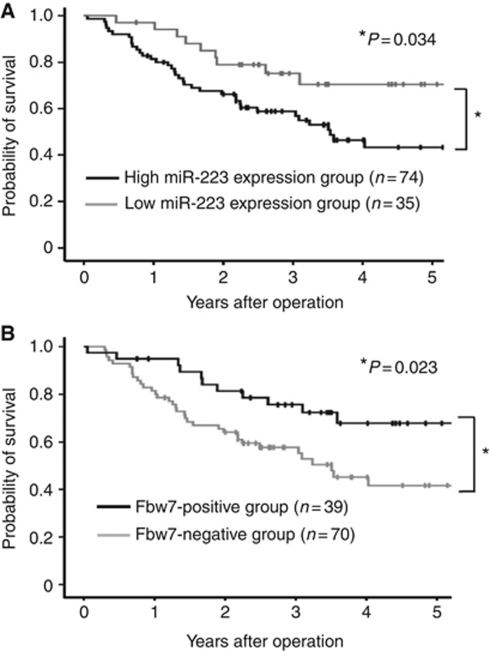
Kaplan–Meier curves according to the miR-223 and FBXW7 status. (**A**) The overall survival curves are presented according to the expression level of miR-223 in ESCC patients. Patients with high miR-223 expression had a poorer prognosis than those with low expression (log-rank (Mantel–Cox) test; *P*=0.034). (**B**) The overall survival curves according to the FBXW7 expression level in ESCC patients. The negative FBXW7 group had a significantly poorer prognosis for 5-year overall survival than the positive group (*P*=0.023).

An analysis of 5-year overall survival demonstrated that the high miR-223 expression group had a significantly poorer prognosis than the low expression group (*P*=0.034; Figure 2A). Similarly, the negative FBXW7 group had a significantly poorer prognosis of 5-year overall survival than the positive group (*P*=0.023; Figure 2B). In a univariate Cox regression analysis, compared with the low miR-223 expression group, the high miR-223 expression group experienced a significantly higher overall mortality (hazard ratio 2.272; 95% confidence interval, 1.099–4.695; *P*=0.027; Table 2). In the univariate analysis, other significant prognostic factors for cancer-specific survival included lymph node metastasis (*P*=0.008), lymphatic invasion (*P*=0.002), and FBXW7 expression (*P*=0.023). In a multivariate Cox regression analysis for overall survival, including age at operation, N status, venous invasion, and miR-223 expression, high miR-223 expression was revealed to be an independent prognostic factor (multivariate hazard ratio 2.425; 95% confidence interval, 1.205–4.878; *P*=0.013; Table 2).

### The clinicopathological significance of FBXW7

We used the H score to evaluate the FBXW7 expression level and defined a final staining score of >50 as positive for FBXW7. Among the 109 ESCC patients, 39 patients (35.8%) showed positive staining for FBXW7. The associations between the patient clinicopathological characteristics and FBXW7 are summarised in Supplementary Table 1. There were no significant differences in the patient clinicopathological characteristics.

### The relationship between FBXW7, and c-Myc and c-Jun in ESCC tissues

**Figure 3:**
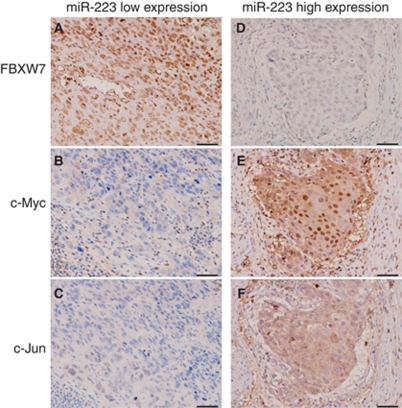
The relationship between the expression of miR-223, and FBXW7, c-Myc, and c-Jun proteins in ESCC patients. (**A**–**C**) In the miR-223-low expression cases, the FBXW7 protein was expressed at a high level, whereas the levels of the c-Myc and c-Jun proteins were below the limit of detection in the same tissue sections. (**D**–**F**) In contrast, in the miR-223-high expression cases, the FBXW7 protein was expressed at a low level, and there was strong expression of the c-Myc and c-Jun proteins. ( × 200 original magnification, scale bar: 50 *μ*m).

We examined the association between the FBXW7 protein expression, and c-Myc and c-Jun protein expression levels in the samples from ESCC patients. When the FBXW7 protein was expressed at high levels, the expression levels of the c-Myc and c-Jun proteins were below the limits of detection in the miR-223 low expression cases (Figures 3A–C). In contrast, in cases with low FBXW7 protein expression, a strong expression of the c-Myc and c-Jun proteins was noted in the cases with high miR-223 expression (Figures 3D–F).

### There is an inverse correlation between miR-223 and *FBXW7 in vitro*

**Figure 4:**
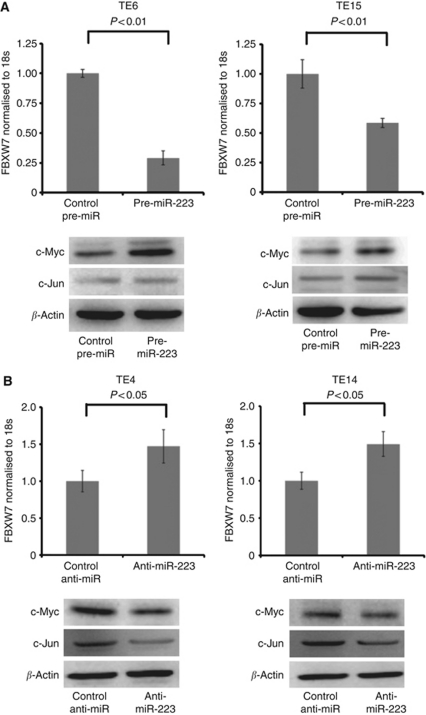
miR-223 gain-of-function and loss-of-function studies in ESCC cell lines. (**A**) The expression of the FBXW7 mRNA was significantly suppressed by transfection of cells with pre-miR-223, as confirmed by qRT–PCR in TE6 and TE15 cells. Furthermore, the expression of the c-Myc and c-Jun proteins was enhanced by treatment with pre-miR-223 as determined by a western blotting analysis. (**B**) In contrast, the FBXW7 mRNA level was significantly increased by transfection of TE4 and TE14 cells with anti-miR-223, and the c-Myc and c-Jun proteins were deregulated.

Across all the eight cell lines tested, there was a significant inverse correlation between the expression levels of miR-223 and *FBXW7* mRNA (Pearson correlation, *r*=−0.855; *P*=0.007; Supplementary Figure 2). TE6 and TE15 cells were used to evaluate the effects of the upregulation of miR-223, and TE4 and TE14 cells were used to examine how the downregulation of miR-223 affected the *FBXW7* expression. *FBXW7* mRNA significantly decreased when cells were transfected with pre-miR-223, compared with those transfected with the negative control (Figure 4A).

Furthermore, we found that the protein expression levels of c-Myc and c-Jun were enhanced after pre-miR-223 treatment by a western blot analysis. In contrast, the TE4 and TE14 cells transfected with anti-miR-223 showed a decrease in the miR-223 expression, compared with the negative control-treated cells. The *FBXW7* mRNA level was significantly increased in the cells transfected with anti-miR-223, compared with those transfected with the negative control, and the protein expression levels of c-Myc and c-Jun were deregulated in these cells (Figure 4B).

## Discussion

In our present study, we found that miR-223 was significantly overexpressed in human ESCC tissue compared with the corresponding normal tissue (*P*<0.001), and that the patients with a high miR-223 expression had a significantly poorer prognosis than those with a low expression (*P*=0.034). We also provide evidence that a negative association exists between the expression of miR-223 and the FBXW7 protein in ESCC patients (Pearson correlation, *r*=−0.336; *P*<0.01), and revealed that the miR-223 expression responds to alterations in the c-Myc and c-Jun protein levels as regulated by the FBXW7 pathway *in vitro.* These findings suggested that the overexpression of miR-223 correlates with the poor prognosis of ESCC, possibly because of repression of the function of the FBXW7 protein.

Loss of FBXW7 function is known to be associated with the dysregulation of several cell cycle regulators, including cyclin E and c-Myc (Welcker *et al*, 2003). In oesophagaeal cancer, amplification and overexpression of these regulators has been thoroughly investigated, and their clinical significance has been reported. Cyclin E, a maintainer of the cell cycle restriction point, is significantly overexpressed in mucosal invasive ESCC compared with normal mucosa (Ohbu *et al*, 2001). The amplification of c-Myc was more frequently found in advanced stages of ESCC than in early stages (Bitzer *et al*, 2003). Therefore, the regulation of FBXW7 may have an important role in the carcinogenesis and progression of ESCC. In this study, there was an inverse correlation between FBXW7, and c-Myc and c-Jun in ESCC samples as indicated by an immunohistochemical analysis. Moreover, an *in vitro* assay demonstrated that there was a decrease in the *FBXW7* expression when miR-223 was overexpressed, which gave rise to an abnormal accumulation of the c-Myc and c-Jun proteins.

miR-223 has been recently reported to have a potential role in tumourigenesis through repressing the function of FBXW7, and the overexpression of miR-223 has been shown to significantly reduce the *FBXW7* mRNA levels, while increasing both the endogenous cyclin E protein and activity levels, as well as genomic instability (Xu *et al*, 2010). Moreover, a recent report identified miR-223 as an E2F1 transcriptional target (Pulikkan *et al*, 2010), and E2F1 and miR-223 comprised an autologous negative feedback loop. These facts indicate that miR-223 is one of the key players in cell cycle regulation at the G1–S transition. In addition, miR-223 has been reported to act as an oncogene in several solid tumours, including gastric, ovarian, and bladder cancers (Gottardo *et al*, 2007; Laios *et al*, 2008; Petrocca *et al*, 2008). Moreover, in gastric cancer, the expression level of miR-223 was reported to be a prognostic marker (Li *et al*, 2010). On the other hand, there has been another report suggesting that miR-223 acts as a tumour suppressor by directly targeting Stathmin1 to stimulate the development and progression of hepatocellular carcinoma (Wong *et al*, 2008). In addition, Li *et al* (2011) revealed the oncogene Artemin to be a target of miR-223 and the overexpression of miR-223 decreased the migration and invasion of oesophageal carcinoma cells. Therefore, on the basis of their findings, miR-223 may have a tumour-suppressor function in oesophageal carcinoma. However, in the current study, on the basis of an investigation of 109 ESCC clinical samples, we showed that miR-223 was significantly overexpressed in the tumour compared with the corresponding normal tissue. We also found that the overexpression of miR-223 correlated with tumour advancement and a poor prognosis. Moreover, in a series of gain-of-function and loss-of-function investigations, we found that these effects may be due to the downregulation of the tumour-suppressor FBXW7, which was targeted by miR-223. The decrease in the expression of *FBXW7* resulting from the overexpression of miR-223 gave rise to the abnormal accumulation of c-Myc and c-Jun proteins. It is well known that one miRNA can regulate many targets and, therefore, it may be possible that the same miRNA may have opposite roles in the progression of cancer in different tissues (Shenouda and Alahari, 2009). As miR-223 also targets other genes, some are oncogenes whereas others are tumour-suppressor genes, further analyses are needed to elucidate the full spectrum of miR-223 functions.

Although we suggested that miR-223 regulates FBXW7, 16 out of 74 samples with high miR-223 expression still showed FBXW7 expression and 12 out of 35 samples with low miR-223 expression did not show FBXW7 as shown in Table 1. The relationship between miR-223 and FBXW7 was therefore not completely inverse. To explain this finding, we speculate that not only miR-223 but also various other mechanisms, have effects on the expression of FBXW7, such as epigenetic transcriptional regulation (Gu *et al*, 2008), the loss of genetic alteration (Iwatsuki *et al*, 2010), the status of *p53* mutation (Yokobori *et al*, 2009), or the regulation by other miRs (miR-25, 27a, 92a) (Xu *et al*, 2010).

In conclusion, the present study at first indicates that a high expression level of miR-223 had a significant adverse impact on the survival of ESCC patients through repression of the function of FBXW7.

## Figures and Tables

**Table 1:** The miR-223 expression and clinicopathological characteristics of the patients

**Factors**	**Total (*n*=109)**	**High (*n*=74)**	**Low (*n*=35)**	***P*-value**
Age (mean±s.d.)		66.0±9.5	65.0±8.4	0.967
				
*Sex*				0.008^*^
Male	90	66	24	
Female	19	8	11	
				
*Histological grade*				0.821
Well	42	28	14	
Moderate, poor, others	67	46	21	
				
*Size*				0.042^*^
<40 mm (small)	50	29	21	
⩾40 mm (large)	59	45	14	
				
*Depth of tumor invasion*				0.030^*^
T1	46	26	20	
T2/3	63	48	15	
				
*Lymph node metastasis*				0.707
Absent	47	31	16	
Present	62	43	19	
				
*Lymphatic invasion*				0.617
Absent	43	28	15	
Present	66	46	20	
				
*Venous invasion*				0.343
Absent	38	28	10	
Present	71	46	25	
				
*Stage*				0.072
I, II	78	49	29	
III, IV	31	25	6	
				
*FBXW7*				0.001^*^
Positive	39	16	23	
Negative	70	58	12	

Abbreviations: moderate=moderately differentiated; poor=poorly differentiated; well=well differentiated.

Note: High miR-223 expression group (*n*=74), miR223 (T)/miR223 (N)⩾1.0; low miR-223 expression group (*n*=35), miR-223 (T)/miR-223 (N)<1.0. ^*^P<0.05.

**Table 2:** The results of univariate and multivariate analyses for overall survival (Cox proportional regression model)

	**Univariate analysis**			**Multivariate analysis**		
**Factors**	**HR**	**95% Cl**	***P*-value**	**HR**	**95% Cl**	***P*-value**
Age (64</65>)	1.559	0.887–2.739	0.123	1.95	1.084–3.506	0.026^*^
Sex (male/female)	0.622	0.265–1.4682	0.276	—	—	—
T1/2,3	1.386	0.719–2.673	0.33	—	—	—
Tumor size (40 mm<, 40 mm⩾)	1.389	0.791–2.438	0.253	—	—	—
Lympha node metastasis (absent/ present)	2.345	1.254–4.385	0.008^*^	2.371	1.250–4.499	0.008^*^
Stage I, II/III, IV	1.947	1.079–3.513	0.027^*^	—	—	—
Venous invasion (absent/present)	1.66	0.892–3.089	0.11	1.42	0.747–2.699	0.285
Lymphatic invasion (absent/present)	2.978	1.513–5.862	0.002^*^	—	—	—
FBXW7 expression (positive/negative)	2.106	1.094–4.057	0.023^*^	—	—	—
miR223 expression (low/high)	2.272	1.099–4.695	0.027^*^	2.425	1.205–4.878	0.013^*^

Abbreviations: CI=confidence interval; HR=hazard ratio.

^*^P<0.05.

## References

[bib1] Ambros V (2004) The functions of animal microRNAs. Nature 431(7006): 350–355 15372042 10.1038/nature02871

[bib2] Bitzer M, Stahl M, Arjumand J, Rees M, Klump B, Heep H, Gabbert HE, Sarbia M (2003) C-myc gene amplification in different stages of oesophageal squamous cell carcinoma: prognostic value in relation to treatment modality. Anticancer Res 23(2B): 1489–1493 12820414

[bib3] Bloomston M, Frankel WL, Petrocca F, Volinia S, Alder H, Hagan JP, Liu CG, Bhatt D, Taccioli C, Croce CM (2007) MicroRNA expression patterns to differentiate pancreatic adenocarcinoma from normal pancreas and chronic pancreatitis. JAMA 297(17): 1901–1908 17473300 10.1001/jama.297.17.1901

[bib4] Bredel M, Bredel C, Juric D, Harsh GR, Vogel H, Recht LD, Sikic BI (2005) Functional network analysis reveals extended gliomagenesis pathway maps and three novel MYC-interacting genes in human gliomas. Cancer Res 65(19): 8679–8689 16204036 10.1158/0008-5472.CAN-05-1204

[bib5] Calin GA, Liu CG, Sevignani C, Ferracin M, Felli N, Dumitru CD, Shimizu M, Cimmino A, Zupo S, Dono M, Dell’Aquila ML, Alder H, Rassenti L, Kipps TJ, Bullrich F, Negrini M, Croce CM (2004) MicroRNA profiling reveals distinct signatures in B cell chronic lymphocytic leukemias. Proc Natl Acad Sci USA 101(32): 11755–11760 15284443 10.1073/pnas.0404432101 PMC511048

[bib6] Croce CM, Calin GA (2005) miRNAs, cancer, and stem cell division. Cell 122(1): 6–7 16009126 10.1016/j.cell.2005.06.036

[bib7] Gottardo F, Liu CG, Ferracin M, Calin GA, Fassan M, Bassi P, Sevignani C, Byrne D, Negrini M, Pagano F, Gomella LG, Croce CM, Baffa R (2007) Micro-RNA profiling in kidney and bladder cancers. Urol Oncol 25(5): 387–392 17826655 10.1016/j.urolonc.2007.01.019

[bib8] Gu Z, Mitsui H, Inomata K, Honda M, Endo C, Sakurada A, Sato M, Okada Y, Kondo T, Horii A (2008) The methylation status of FBXW7 beta-form correlates with histological subtype in human thymoma. Biochem Biophys Res Commun 377(2): 685–688 18938137 10.1016/j.bbrc.2008.10.047

[bib9] Hagedorn M, Delugin M, Abraldes I, Allain N, Belaud-Rotureau MA, Turmo M, Prigent C, Loiseau H, Bikfalvi A, Javerzat S (2007) FBXW7/hCDC4 controls glioma cell proliferation *in vitro* and is a prognostic marker for survival in glioblastoma patients. Cell Div 2: 9 17326833 10.1186/1747-1028-2-9 PMC1819378

[bib10] Hiyoshi Y, Kamohara H, Karashima R, Sato N, Imamura Y, Nagai Y, Yoshida N, Toyama E, Hayashi N, Watanabe M, Baba H (2009) MicroRNA-21 regulates the proliferation and invasion in esophageal squamous cell carcinoma. Clin Cancer Res 15(6): 1915–1922 19276261 10.1158/1078-0432.CCR-08-2545

[bib11] Ibusuki M, Yamamoto Y, Shinriki S, Ando Y, Iwase H (2011) Reduced expression of ubiquitin ligase FBXW7 mRNA is associated with poor prognosis in breast cancer patients. Cancer Sci 102(2): 439–445 21134077 10.1111/j.1349-7006.2010.01801.x

[bib12] Inuzuka H, Shaik S, Onoyama I, Gao D, Tseng A, Maser RS, Zhai B, Wan L, Gutierrez A, Lau AW, Xiao Y, Christie AL, Aster J, Settleman J, Gygi SP, Kung AL, Look T, Nakayama KI, DePinho RA, Wei W (2011) SCF(FBW7) regulates cellular apoptosis by targeting MCL1 for ubiquitylation and destruction. Nature 471(7336): 104–109 21368833 10.1038/nature09732 PMC3076007

[bib13] Iwatsuki M, Mimori K, Ishii H, Yokobori T, Takatsuno Y, Sato T, Toh H, Onoyama I, Nakayama KI, Baba H, Mori M (2010) Loss of FBXW7, a cell cycle regulating gene, in colorectal cancer: clinical significance. Int J Cancer 126(8): 1828–1837 19739118 10.1002/ijc.24879

[bib14] Laios A, O’Toole S, Flavin R, Martin C, Kelly L, Ring M, Finn SP, Barrett C, Loda M, Gleeson N, D’Arcy T, McGuinness E, Sheils O, Sheppard B, O' Leary J (2008) Potential role of miR-9 and miR-223 in recurrent ovarian cancer. Mol Cancer 7: 35 18442408 10.1186/1476-4598-7-35 PMC2383925

[bib15] Li S, Li Z, Guo F, Qin X, Liu B, Lei Z, Song Z, Sun L, Zhang HT, You J, Zhou Q (2011) miR-223 regulates migration and invasion by targeting Artemin in human esophageal carcinoma. J Biomed Sci 18: 24 21453483 10.1186/1423-0127-18-24 PMC3080798

[bib16] Li X, Zhang Y, Ding J, Wu K, Fan D (2010) Survival prediction of gastric cancer by a seven-microRNA signature. Gut 59(5): 579–585 19951901 10.1136/gut.2008.175497

[bib17] Mao JH, Kim IJ, Wu D, Climent J, Kang HC, DelRosario R, Balmain A (2008) FBXW7 targets mTOR for degradation and cooperates with PTEN in tumor suppression. Science 321(5895): 1499–1502 18787170 10.1126/science.1162981 PMC2849753

[bib18] Meister G (2007) miRNAs get an early start on translational silencing. Cell 131(1): 25–28 17923084 10.1016/j.cell.2007.09.021

[bib19] Nakayama KI, Nakayama K (2006) Ubiquitin ligases: cell-cycle control and cancer. Nat Rev Cancer 6(5): 369–381 16633365 10.1038/nrc1881

[bib20] Ohbu M, Kobayashi N, Okayasu I (2001) Expression of cell cycle regulatory proteins in the multistep process of oesophageal carcinogenesis: stepwise over-expression of cyclin E and p53, reduction of p21(WAF1/CIP1) and dysregulation of cyclin D1 and p27(KIP1). Histopathology 39(6): 589–596 11903577 10.1046/j.1365-2559.2001.01279.x

[bib21] Petrocca F, Visone R, Onelli MR, Shah MH, Nicoloso MS, de Martino I, Iliopoulos D, Pilozzi E, Liu CG, Negrini M, Cavazzini L, Volinia S, Alder H, Ruco LP, Baldassarre G, Croce CM, Vecchione A (2008) E2F1-regulated microRNAs impair TGFbeta-dependent cell-cycle arrest and apoptosis in gastric cancer. Cancer Cell 13(3): 272–286 18328430 10.1016/j.ccr.2008.02.013

[bib22] Pulikkan JA, Dengler V, Peramangalam PS, Peer Zada AA, Muller-Tidow C, Bohlander SK, Tenen DG, Behre G (2010) Cell-cycle regulator E2F1 and microRNA-223 comprise an autoregulatory negative feedback loop in acute myeloid leukemia. Blood 115(9): 1768–1778 20029046 10.1182/blood-2009-08-240101 PMC2832809

[bib23] Schetter AJ, Leung SY, Sohn JJ, Zanetti KA, Bowman ED, Yanaihara N, Yuen ST, Chan TL, Kwong DL, Au GK, Liu CG, Calin GA, Croce CM, Harris CC (2008) MicroRNA expression profiles associated with prognosis and therapeutic outcome in colon adenocarcinoma. JAMA 299(4): 425–436 18230780 10.1001/jama.299.4.425 PMC2614237

[bib24] Shenouda SK, Alahari SK (2009) MicroRNA function in cancer: oncogene or a tumor suppressor? Cancer Metastasis Rev 28(3–4): 369–378 20012925 10.1007/s10555-009-9188-5

[bib25] Ueda T, Volinia S, Okumura H, Shimizu M, Taccioli C, Rossi S, Alder H, Liu CG, Oue N, Yasui W, Yoshida K, Sasaki H, Nomura S, Seto Y, Kaminishi M, Calin GA, Croce CM (2010) Relation between microRNA expression and progression and prognosis of gastric cancer: a microRNA expression analysis. Lancet Oncol 11(2): 136–146 20022810 10.1016/S1470-2045(09)70343-2 PMC4299826

[bib26] Welcker M, Clurman BE (2008) FBW7 ubiquitin ligase: a tumour suppressor at the crossroads of cell division, growth and differentiation. Nat Rev Cancer 8(2): 83–93 18094723 10.1038/nrc2290

[bib27] Welcker M, Singer J, Loeb KR, Grim J, Bloecher A, Gurien-West M, Clurman BE, Roberts JM (2003) Multisite phosphorylation by Cdk2 and GSK3 controls cyclin E degradation. Mol Cell 12(2): 381–392 14536078 10.1016/s1097-2765(03)00287-9

[bib28] Wertz IE, Kusam S, Lam C, Okamoto T, Sandoval W, Anderson DJ, Helgason E, Ernst JA, Eby M, Liu J, Belmont LD, Kaminker JS, O’Rourke KM, Pujara K, Kohli PB, Johnson AR, Chiu ML, Lill JR, Jackson PK, Fairbrother WJ, Seshagiri S, Ludlam MJ, Leong KG, Dueber EC, Maecker H, Huang DC, Dixit VM (2011) Sensitivity to antitubulin chemotherapeutics is regulated by MCL1 and FBW7. Nature 471(7336): 110–114 21368834 10.1038/nature09779

[bib29] Wong QW, Lung RW, Law PT, Lai PB, Chan KY, To KF, Wong N (2008) MicroRNA-223 is commonly repressed in hepatocellular carcinoma and potentiates expression of Stathmin1. Gastroenterology 135(1): 257–269 18555017 10.1053/j.gastro.2008.04.003

[bib30] Xu Y, Sengupta T, Kukreja L, Minella AC (2010) MicroRNA-223 regulates cyclin E activity by modulating expression of F-box and WD-40 domain protein 7. J Biol Chem 285(45): 34439–34446 20826802 10.1074/jbc.M110.152306 PMC2966058

[bib31] Yanaihara N, Caplen N, Bowman E, Seike M, Kumamoto K, Yi M, Stephens RM, Okamoto A, Yokota J, Tanaka T, Calin GA, Liu CG, Croce CM, Harris CC (2006) Unique microRNA molecular profiles in lung cancer diagnosis and prognosis. Cancer Cell 9(3): 189–198 16530703 10.1016/j.ccr.2006.01.025

[bib32] Yokobori T, Mimori K, Iwatsuki M, Ishii H, Onoyama I, Fukagawa T, Kuwano H, Nakayama KI, Mori M (2009) p53-Altered FBXW7 expression determines poor prognosis in gastric cancer cases. Cancer Res 69(9): 3788–3794 19366810 10.1158/0008-5472.CAN-08-2846

[bib33] Zamore PD, Haley B (2005) Ribo-gnome: the big world of small RNAs. Science 309(5740): 1519–1524 16141061 10.1126/science.1111444

